# Multidisciplinary care of obese children and adolescents for one year reduces ectopic fat content in liver and skeletal muscle

**DOI:** 10.1186/s12887-015-0513-6

**Published:** 2015-12-30

**Authors:** Cilius Esmann Fonvig, Elizaveta Chabanova, Johanne Dam Ohrt, Louise Aas Nielsen, Oluf Pedersen, Torben Hansen, Henrik S. Thomsen, Jens-Christian Holm

**Affiliations:** The Children’s Obesity Clinic, Department of Pediatrics, Copenhagen University Hospital Holbæk, 4300 Holbæk, Denmark; The Novo Nordisk Foundation Center for Basic Metabolic Research, Section of Metabolic Genetics, Faculty of Medical and Health Sciences, University of Copenhagen, 2100 Copenhagen Ø, Denmark; Department of Diagnostic Radiology, Copenhagen University Hospital Herlev, 2730 Herlev, Denmark; University of Copenhagen, Faculty of Medical and Health Sciences, 2200 Copenhagen N, Denmark

**Keywords:** Pediatric Obesity, Magnetic Resonance Spectroscopy, Skeletal Muscle, Non-alcoholic Fatty Liver Disease, Dyslipidemia, Glucose Metabolic Disorders, Child, Adolescent

## Abstract

**Background:**

Ectopic fat deposition in liver and skeletal muscle tissue is related to cardiovascular disease risk and is a common metabolic complication in obese children. We evaluated the hypotheses of ectopic fat in these organs could be diminished following 1 year of multidisciplinary care specialized in childhood obesity, and whether this reduction would associate with changes in other markers of metabolic function.

**Methods:**

This observational longitudinal study evaluated 40 overweight children and adolescents enrolled in a multidisciplinary treatment protocol at the Children’s Obesity Clinic, Holbæk, Denmark. The participants were assessed by anthropometry, fasting blood samples (HbA1c, glucose, insulin, lipids, and biochemical variables of liver function), and liver and muscle fat content assessed by magnetic resonance spectroscopy at enrollment and following an average of 12.2 months of care. Univariate linear regression models adjusted for age, sex, treatment duration, baseline degree of obesity, and pubertal developmental stage were used for investigating possible associations.

**Results:**

The standard deviation score (SDS) of baseline median body mass index (BMI) was 2.80 (range: 1.49–3.85) and the median age was 14 years (10–17). At the end of the observational period, the 40 children and adolescents (21 girls) significantly decreased their BMI SDS, liver fat, muscle fat, and visceral adipose tissue volume. The prevalence of hepatic steatosis changed from 28 to 20 % (*p* = 0.26) and the prevalence of muscular steatosis decreased from 75 to 45 % (*p* = 0.007).

Changes in liver and muscle fat were independent of changes in BMI SDS, baseline degree of obesity, duration of treatment, age, sex, and pubertal developmental stage.

**Conclusions:**

A 1-year multidisciplinary intervention program in the setting of a childhood obesity outpatient clinic confers a biologically important reduction in liver and muscle fat; metabolic improvements that are independent of the magnitude of concurrent weight loss.

**Trial registration:**

ClinicalTrials.gov registration number: NCT00928473, the Danish Childhood Obesity Biobank. Registered June 25, 2009.

**Electronic supplementary material:**

The online version of this article (doi:10.1186/s12887-015-0513-6) contains supplementary material, which is available to authorized users.

## Background

Hepatic and muscular steatosis are common metabolic abnormalities in obese children [1, 2]. Childhood onset accumulation of ectopic fat in liver and skeletal muscle indicates an increased cardiovascular disease risk including dyslipidemia and insulin resistance [3–8], the latter being a metabolic abnormality that precedes the development of type 2 diabetes [9].

Several methods can assess the content of ectopic lipid accumulation, including computed tomography, ultrasound, tissue biopsies, proton magnetic resonance spectroscopy (MRS), and magnetic resonance imaging (MRI) [10]. The non-invasive and non-ionizing MRS is considered gold standard in muscle lipid quantification [10] and may in the future replace liver biopsies as the gold standard in the quantification of liver fat, although it is not providing information regarding histological alterations [10–12].

Studies on treatment of ectopic fat accumulation in childhood mainly address hepatic steatosis and the existing literature proposes lifestyle intervention and weight loss as the therapeutics of choice [13, 14]. Despite the increasing prevalence in pediatric hepatic steatosis, a targeted treatment strategy of this condition has yet to be established, and the potential future increase in a broad array of liver and muscular steatosis-related morbidities calls for further progress in this field of research [13, 14].

The outlined multidisciplinary care protocol to combat obesity has previously been reported to associate with reduction of body mass index (BMI) standard deviation score (SDS) in a study of 492 overweight and obese children and youths [15] and with improved fasting serum lipid profiles in a study of 240 overweight and obese children and youths [16].

The objective of this 1-year observational study was to investigate the impact of the multidisciplinary care protocol practiced in our outpatient clinic of childhood obesity with a focus on changes in ectopic deposition of fat in the liver and skeletal muscles. We hypothesized that ectopic fat in these organs could be reduced following 1 year of childhood obesity treatment, and that this reduction would associate with changes in other markers of metabolic function.

## Methods

### Study population

From August 2009 to October 2014, 1406 overweight children and adolescents were enrolled in treatment at The Children’s Obesity Clinic, Department of Pediatrics, Copenhagen University Hospital Holbæk, Denmark [15]. Of these, 398 were offered an MR-scan at the time of treatment start, and hereof 92 were subsequently offered a follow-up MR-scan after 1 year of treatment. The inclusion criteria were i) 8–18 years of age at enrollment, ii) enrollment in childhood obesity treatment, iii) each of the two MR assessments of liver and muscle lipid accumulation (at baseline and at follow-up) should have concomitant anthropometric and biochemical measures within a 60 days period, and iv) a baseline BMI SDS above 1.28, which corresponds to the 90^th^ percentile according to Danish age- and sex-adjusted references [17]. The exclusion criteria were i) a body weight above 135 kg, which was the maximum capacity of the MR scanner, ii) inability to remain quiet in the MR machine during the 45 minutes scan time, iii) presence of other liver diseases, iv) development of type 2 diabetes mellitus during the treatment period, or v) an alcohol consumption of more than 140 g/week.

### Treatment

The Children’s Obesity Clinic is a chronic care, multidisciplinary, best-practice, hospital-based, outpatient, childhood obesity treatment center involving a staff core of pediatricians, dieticians, nurses, psychologists, social workers, secretaries, and research technicians [15]. Some baseline examinations are performed as in-patient admissions. Children and adolescents are referred for treatment from their general practitioners, school- and community based doctors, or pediatricians (at hospitals or private practices) from all over Denmark. At inclusion, a pediatrician sees the child and family for 1 hour, where the medical history and a physical examination of the child are performed. At this visit the child and family are introduced to the treatment protocol, which is a family-centered approach involving behavior-modifying techniques, where the child and family receive an individually tailored and thorough plan of lifestyle advices [15]. This plan addresses sugar and fat intake, sources of nutrition, activity, inactivity, psychosocial capabilities, disturbed eating behaviors, sleeping disorders, hygiene, allowances, and more [15]. The child and family are scheduled to consult a pediatrician on an annual basis and a pediatric nurse, dietician, and/or psychologist as needed. The treatment plan is evaluated at every visit. Each family is on average seen in the clinic every 6.5 weeks, with a mean of 5.4 hours of health professional time spent on each patient per year [15].

The treatment protocol for the Children’s Obesity Clinic is described in detail by Holm *et al.* [15], and the appendix “Information to the readers” is furthermore available from the authors.

### Anthropometry

Body weight was measured to the nearest 0.1 kg on a Tanita digital medical scale (WB-100 MA; Tanita Corp., Tokyo, Japan). Height was measured to the nearest 1 mm by a stadiometer. Weight and height were measured with bare feet in underwear or light indoor clothing. BMI was calculated as weight divided by height squared (kg/m^2^). The BMI SDS was calculated by the LMS method by converting BMI into a normal distribution by sex and age using the median coefficient of variation and a measure of the skewness [18] based on the Box-Cox power plot based on Danish BMI charts [17].

### Pubertal development

The pubertal stage was determined at baseline by a trained pediatrician using the classification of Tanner [19]. In boys, the developmental stages of pubic hair and genitals were determined, and testes size was determined by an orchidometer. In girls, the developmental stages of breasts and pubic hair were determined.

### MR spectroscopy and imaging

MR measurements were performed on a 3.0 T MR imaging system (Achieva, Philips Medical Systems, Best, The Netherlands) using a SENSE cardiac coil and the data post processing was performed by an experienced MR physicist. The participants were examined in the supine position. Liver fat content (LFC) and muscle fat content (MFC) were measured by MRS. MFC was measured in the psoas muscle. Visceral adipose tissue (VAT) and subcutaneous adipose tissue (SAT) volumes were measured by MRI, assessed from a transverse slice of 10 mm thickness at the level of the third lumbar vertebra. The details of the applied methodology of MRI and MRS have previously been described [1, 2].

Hepatic steatosis was defined as an LFC >5 % [20] and muscular steatosis was defined as an MFC >5 % [2].

### Blood sampling

Blood samples were drawn from an antecubital vein between 7 a.m. and 9 a.m. after an overnight fast. If required, an anesthetic cream was applied one hour before venipuncture. The biochemical analyses of plasma concentrations of glucose and serum concentrations of triglycerides, total cholesterol, high density lipoprotein (HDL) cholesterol, alanine transaminase, and gamma-glutamyl transferase were performed on a Dimension Vista® 1500 analyzer (Siemens, Munich, Germany). Plasma glucose samples and the serum samples of triglycerides, cholesterol fractions, and biochemical variables of liver function were stored at room temperature for less than 30 min after sampling before being centrifuged at four degrees Celsius. Plasma glucose samples were collected in tubes containing fluoride. The biochemical analyses of serum insulin concentrations were performed on a Cobas® 6000 analyzer (F. Hoffmann-La Roche Ltd, Basel, Switzerland) and stored at room temperature for 30–60 min after sampling before being centrifuged at four degrees Celsius. Analyses of all plasma and serum samples were performed immediately after being centrifuged. Insulin samples were collected in a tube containing serum separating gel. The biochemical analyses of whole blood glycosylated hemoglobin (HbA1c) were performed on a Tosoh high-performance liquid chromatography G8 analyzer (Tosoh Corporation, Tokyo, Japan). The low density lipoprotein (LDL) cholesterol concentration was calculated as: Total cholesterol – (triglycerides × 0.45) + HDL cholesterol. The Non-HDL cholesterol concentration was calculated as: Total cholesterol – HDL cholesterol.

### Statistical analysis

Wilcoxon signed rank test was used to analyze differences in continuous variables between groups and to analyze estimations of differences from baseline to follow-up and the corresponding nonparametric confidence intervals (CI). The differences in fractions of steatosis were analyzed by McNemar’s Test for paired categorical data. Associations were investigated by univariate linear regression models adjusted for age, sex, treatment duration, baseline degree of obesity, and pubertal developmental stage. The linear regression analyses were based on the logarithmically transformed baseline and follow-up values. *P*-values were not adjusted for multiple hypothesis testing and the level of significance was set at *p* <0.05. Statistical analyses were performed using “R” statistical software version 3.1.2 (http://www.r-project.org).

### Ethical aspects

Informed written consent was obtained from the parents of patients younger than 18 years and from patients of 18 years of age. The study was approved by the Ethics Committee of Region Zealand, Denmark (SJ-104) and the Danish Data Protection Agency (REG-06-2014) and is registered at ClinicalTrials.gov (NCT00928473). This study has been reported in line with the STROBE guidelines (Additional file 1).

## Results

Of the 92 who were offered two MRS assessments, 40 overweight and obese children and adolescents fulfilled the inclusion criteria. Beside these, five patients were excluded because they had a body weight >135 kg, one patient was excluded from the study because of the development of type 2 diabetes mellitus during the study period, and 46 children and adolescents fulfilled all criteria except for having blood samples drawn within the 60 days period of the MR assessment. None were excluded due to an inability to stay quiet during the scan time, other liver diseases, or an alcohol consumption of more than 140 g/week. The group not complying with the blood sample criterion were comparable to the 40 included children and adolescents in regards to BMI SDS, VAT, SAT, and liver fat content before and after treatment (data not shown). The 40 overweight/obese children and adolescents (21 girls) had a baseline median BMI SDS of 2.80 (range 1.49–3.85) and a median age of 13.7 years (10.0–16.8). MRS, MRI, and concomitant anthropometric and biochemical measures were performed on all study participants at baseline and after a median of 12 months of follow-up (Table 1). The time between the MR scan and the biochemical measures was a median of 10 days (range: 0–58) at baseline and 10 days (1–59) at follow-up. Blood samples were performed within 30 days from the anthropometric measures (median: 12 days), and the time between the MR scan and the anthropometric measures was a median of 14 days (range: 0–56) at baseline and 17 days (1–53) at follow-up.

**Table 1:** Characteristics of the 40 (21 girls) overweight children and adolescents

	Baseline	Follow-up	*p*
Age, *years*	13.7 (10.0–16.8)	14.6 (10.9–17.8)	<0.0001***
BMI SDS	2.80 (1.49–3.85)	2.56 (0.18–4.68)	0.001**
VAT, *cm* ^*3*^	83 (21–361)	73 (15–396)	0.01*
SAT, *cm* ^*3*^	282 (97–518)	262 (74–527)	0.14
LFC, *%*	3.0 (0.5–67.0)	3.0 (0.5–32.0)	0.01*
Hepatic steatosis, *fraction*	28 % (11/40)	20 % (8/40)	0.26
MFC, *%*	7.4 (1.2–26.3)	4.8 (0.5–39.6)	0.01*
Muscle steatosis, *fraction*	75 % (30/40)	45 % (18/40)	0.007**
Triglyceride, *mmol/l*	0.9 (0.2–2.3)	1.0 (0.3–2.0)	0.78
HDL cholesterol, *mmol/l*	1.2 (0.7–1.7)	1.2 (0.8–2.1)	0.03*
LDL cholesterol, *mmol/l*	2.3 (1.1–4.2)	2.4 (1.0–3.8)	0.02*
Non-HDL cholesterol, *mmol/l*	2.8 (1.2–4.7)	2.8 (1.2–4.4)	0.02*
Plasma glucose, *mmol/l*	5.1 (4.2–6.2)	5.1 (4.4–5.9)	0.42
Serum insulin, *pmol/l*	83 (11–271)	87 (14–226)	0.99
HbA1c, *mmol/l*	35 (28–42)	34 (26–40)	0.04*
ALT, *U/l*	22 (11–126)	22 (10–69)	0.67
GGT, *U/l*	18 (5–134)	16 (9–33)	0.72

Data are medians (range) due to a non-normal distribution

*ALT* alanine transaminase; *BMI* body mass index; *GGT* gamma-glutamyl transferase; *HDL* high density lipoprotein; *HbA1c* glycosylated hemoglobin; *IMCL* intramyocellular lipid content; *LDL* low density lipoprotein; *LFC* liver fat content; *MFC* muscle fat content; *SAT* subcutaneous adipose tissue volume; *SDS* standard deviation score; *VAT* visceral adipose tissue volume

*P* value for group differences: *** *p* <0.001; ** *p* <0.01; * *p* <0.05

The 1406 children and adolescents included in treatment were 1.5 years younger (95 % CI: 0.6–2.5, *p* = 0.001) than the 40 included children and adolescents, but comparable in baseline BMI-SDS (difference: 0.1, CI 95 %: −0.1–0.3, *p* = 0.23).

### Treatment

The characteristics of the 40 overweight and obese children and adolescents at baseline and follow-up are shown in Table 1. After an average of 12.2 months (95 % CI: 11.9–13.1) of treatment, BMI SDS was reduced by 0.23 (95 % CI: 0.10–0.44, *p* = 0.001) accompanied by reductions in liver fat percentage (1.0, 95 % CI: 0.3–3.6, *p* = 0.01), muscle fat percentage (2.4, 95 % CI: 0.7–4.0, *p* = 0.01), and VAT volume (14 cm^3^, 95 % CI: 3–27, *p* = 0.01). Furthermore, we observed reductions in concentrations of whole blood HbA1c by 1.0 mmol/mol (95 % CI: 0.0–2.0, *p* = 0.04), fasting serum levels of LDL cholesterol by 0.2 mmol/l (95 % CI: 0.0–0.4, *p* = 0.02), and non-HDL cholesterol by 0.2 mmol/l (95 % CI: 0.0–0.4, *p* = 0.02), and an increase in fasting serum HDL cholesterol concentration of 0.1 mmol/l (95 % CI: 0.0–0.2, *p* = 0.03).

**Fig. 1:**
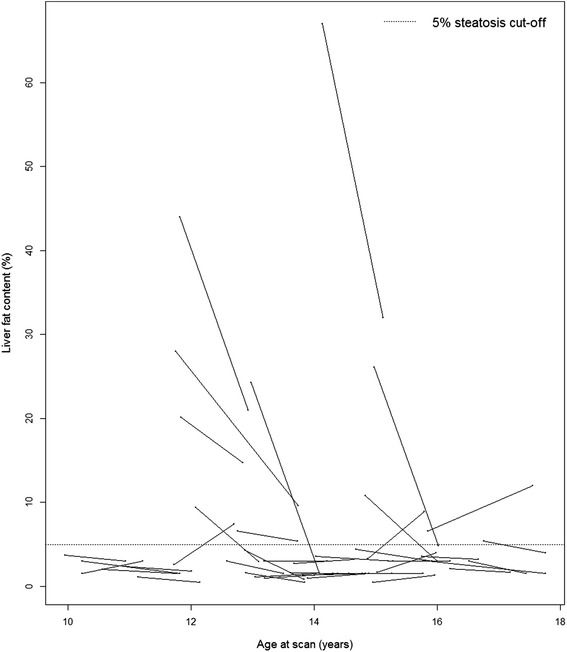
Liver Fat Development during Treatment. The development of liver fat content for the individual study participants during an average follow-up of 12.2 months

**Fig. 2:**
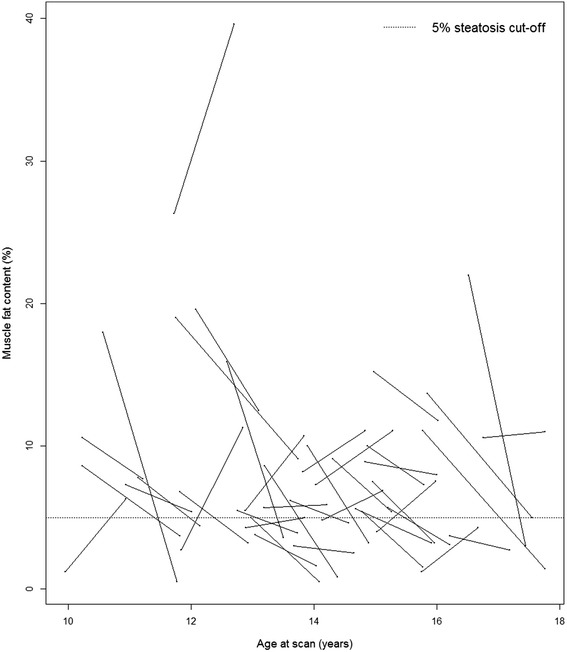
Muscle Fat Development during Treatment. The development of muscle fat content for the individual study participants during an average follow-up of 12.2 months

The individual treatment responses on levels of liver and muscle fat are shown in the Figs. 1 and 2, respectively. At baseline, the prevalence of hepatic steatosis was 28 %; a fraction that was 20 % at follow-up (*p* = 0.26) (Table 1). Two of the 29 (7 %) study patients without hepatic steatosis at baseline exhibited hepatic steatosis at follow-up, while five of the 11 (45 %) with hepatic steatosis at baseline exhibited no hepatic steatosis at follow-up. Muscular steatosis was reduced from 75 % at baseline to 45 % at follow-up (*p* = 0.007) (Table 1). Four of the ten (40 %) patients without muscular steatosis at baseline exhibited muscular steatosis at follow-up, while 16 of the 30 (53 %) with muscular steatosis at baseline exhibited no muscular steatosis at follow-up.

We observed no significant changes in fasting concentrations of plasma triglyceride, plasma glucose, serum insulin, or biochemical variables of liver function (Table 1).

### Changes in liver fat content

Changes in LFC, adjusted for the baseline level of LFC, age, sex, treatment duration, baseline degree of obesity, and pubertal developmental stage, associated positively with changes in MFC (*p* = 0.045) and inversely with baseline levels of liver fat (*p* = 0.001). Changes in LFC were not significantly associated with baseline levels of or changes in BMI SDS (*p* = 0.30, *p* = 0.57), VAT (*p* = 0.47, *p* = 0.45), SAT (*p* = 0.27, *p* = 0.21), or fasting concentrations of triglycerides (*p* = 0.49, *p =* 0.78), HDL cholesterol (*p* = 0.83, *p =* 0.62), LDL cholesterol (*p* = 0.67, *p =* 0.06), non-HDL cholesterol (*p* = 0.63, *p =* 0.07), plasma glucose (*p* = 0.66, *p* = 0.67), serum insulin (*p* = 0.07, *p* = 0.12), HbA1c (*p* = 0.61, *p* = 0.50), alanine transaminase (*p* = 0.87, *p =* 0.16), or gamma-glutamyl transferase (*p* = 0.83, *p* = 0.16).

While the group not exhibiting hepatic steatosis at baseline maintained the degree of LFC (median change: −0.1 %, (interquartile range: −0.7; 0.5)), the group exhibiting hepatic steatosis decreased the LFC by a median −7.8 % (−22.0; −3.4) (p-value for difference: *p* = 0.0003).

### Changes in muscle fat content

Changes in MFC, adjusted for the baseline level of MFC, age, sex, treatment duration, baseline degree of obesity, and pubertal developmental stage, associated positively with changes in VAT (*p* = 0.001) and inversely with baseline levels of MFC (*p* = 0.0005). Changes in MFC were not significantly associated with baseline levels of or changes in BMI SDS (*p* = 0.17, *p* = 0.36), LFC (*p* = 0.25, *p* = 0.47), SAT (*p* = 0.15, *p* = 0.57), or fasting concentrations of triglycerides (*p* = 0.11, *p =* 0.86), HDL cholesterol (*p* = 0.85, *p =* 0.45), LDL cholesterol (*p* = 0.11, *p =* 0.28), non-HDL cholesterol (*p* = 0.07, *p =* 0.24), plasma glucose (*p* = 0.66, *p* = 0.37), serum insulin (*p* = 0.53, *p* = 0.21), or HbA1c (*p* = 0.06, *p* = 0.52).

While the group not exhibiting muscular steatosis at baseline tended to increase the degree of MFC (median change: 2.1 %, (interquartile range: −0.5; 3.6)), the group exhibiting muscular steatosis decreased the MFC by a median −3.4 % (−7.0; −1.6) (p-value for difference: *p* = 0.74).

Only the inverse associations between the change in and the baseline level of both LFC and MFC remained significant after adjusting for multiple testing ad modum Benjamini & Hochberg (data not shown).

## Discussion

This 1-year multidisciplinary intervention program associated with a biologically important reduction in liver and muscle fat as assessed by magnetic resonance measures. Comparable findings of concomitant reductions in BMI SDS, MRI-measured liver fat, and waist circumference (as a surrogate measure of visceral fat) have been reported in a 1-year nutrition-behavior intervention study of 26 obese children with an age of 6–14 years [21]. The present study extends these findings by reporting reductions in ectopic fat content in liver and muscle independent of the magnitude of weight loss. In a 12-week exercise intervention study of 15 obese and 14 lean post-pubertal adolescents, van der Heijden *et al.* [22] observed reductions in MRS-measured liver fat, but without reductions in intramyocellular lipids (IMCL) or BMI SDS suggesting the beneficial effect of longer treatment periods, as observed in the present study.

In two multidisciplinary childhood obesity treatment programs of 6 and 12 months duration, respectively, Koot *et al.* [23] in a study of 144 children and adolescents and Reinehr *et al.* [24] in a study of 109 children and adolescents reported reductions in ultrasound-measured LFC and BMI SDS. Compared to MRS, ultrasonographic longitudinal studies have some limitations since they provide less precise and reproducible quantitative information and have great inter- and intraobserver variability [25]. Although both studies used a single experienced observer, Koot *et al.* [23] still reported an intraobserver agreement as low as 57 %, whereas Reinehr *et al.* [24] did not report observer variability.

A study on seven adults reported a reduction in MRS-measured IMCL during 9 weeks of dietary weight loss intervention [26], while a 12-week dietary weight loss intervention of 13 non-diabetic obese adults found no reductions in MRS-measured IMCL [27]. These differences might reflect a considerable variability in the accumulation of fat in muscle tissue, which is also suggested in the 40 % of the present study participants who shifted from no muscular steatosis to muscular steatosis, although we observed a significant majority of the patients shifting from muscular steatosis to no steatosis (Fig. 2). Furthermore, in ten obese adults, a 6 months weight loss intervention reduced MRS-measured IMCL in the mainly glycolytic tibialis muscle [28], but not in the mainly oxidative soleus muscle, despite that glycolytic muscles, including the psoas muscle, generally contain lower amounts of fat as compared to oxidative muscles [29, 30].

### Glucose metabolism

Associations between the accumulation of fat in skeletal muscle and dysregulation of the glucose metabolism have been reported in both cross-sectional [31] and longitudinal studies [26].

In a weight loss study of seven overweight adults undergoing dietary intervention alone compared to nine overweight adults undergoing combined dietary and exercise intervention, Toledo *et al.* reported comparable changes in weight loss and insulin sensitivity in the two groups, while biopsy-proven IMCL was reduced only in the dietary intervention group [32]. This suggests that muscle lipid accumulation is independent of insulin sensitivity, which is also suggested in the study by van der Heijden *et al.* where insulin sensitivity improved without reductions in IMCL [22].

The relationship between fatty liver and elevated fasting circulating levels of glucose and insulin has been reported in cross-sectional studies [4, 5]. In the aforementioned intervention study by van der Heijden *et al,* reductions in liver and visceral fat correlated with reductions in circulating insulin concentrations in the group of obese adolescents [22]. Several longitudinal studies of reductions in LFC assessed by ultrasound have shown concomitant improvements in glucose metabolism in 144, 84, 71, and 20 children and adolescents, respectively [23, 33–35], suggesting a positive association between LFC and insulin resistance. In contrast, Pozzato *et al.* [21] and Reinehr *et al.* [24] did not observe any associations between changes in liver fat and changes in fasting glucose or insulin levels, despite comparable sample sizes. In the present study, no reductions were seen in either fasting insulin or glucose, despite improvements in a range of other metabolic markers. This is most likely due to a majority of study participants undergoing puberty during the treatment period, and the transitory physiological insulin resistance (worsening glucose metabolism) in the pubertal period that potentially overshadows any improvements resulting from the treatment [36]. Nonetheless, we did observe reductions in HbA1c in the present study.

### Lipid metabolism

Cross-sectional studies in children and adolescents have reported positive associations between serum lipid profiles and steatosis in liver [37] and muscle [38]. Longitudinal pediatric studies have shown relationships between lipid profiles and hepatic steatosis [23] and liver fibrosis [33] - a complication to hepatic steatosis - although none of these associations remained significant in multivariate analyses [23, 33]. In two longitudinal studies on concomitant changes in MFC and serum lipid variables in adults, no significant associations have been reported [26, 27]. Although improvements in the general serum lipid profile were observed in the present study, no significant association to ectopic fat in liver and muscle were observed.

### Relationship between the ectopic fat depots

The deposition of lipids in the ectopic fat depots is thought to take place when the capacity of the subcutaneous adipose tissue is exceeded [3]. Positive correlations have previously been reported between LFC and VAT [1, 27, 28], MFC and VAT [2], and between LFC and MFC [5], suggesting that storage and mobilization of lipids in these ectopic depots are interconnected. These findings are in line with results in the present study, except for the lack of association between LFC and VAT. In the present study, we furthermore observed that the changes in ectopic fat content in liver and muscle were inversely associated with their respective baseline levels, suggesting that individuals with a higher level of ectopic tissue fat at the baseline exhibited greater reductions in ectopic fat content during treatment. This observation may be (partly) explained by the phenomenon ‘regression towards the mean’.

### Biochemical markers of liver function

Changes in LFC have been positively associated with changes in alanine transaminase and gamma-glutamyl transferase in childhood obesity treatment [39] of a comparable sample size to the present study. Even though childhood obesity has been linked to fatty liver [40] and elevated concentrations of liver enzymes [41], the measures of variables serving as proxies for liver function may deviate from and potentially underestimate pathological histological alterations in the liver [42]. In line with the latter, we observed no relationships between liver fat changes and baseline or follow-up levels of liver function markers.

### Strengths and limitations

A strength of the present protocol is the relatively high number of participants with simultaneous MRS-assessed fat content in liver and muscle and concomitant measures of anthropometrics and pertinent fasting biochemical blood variables measured before and after a 1-year treatment period in a best-practice based multidisciplinary regimen focused at combating childhood obesity.

One of the major limitations of our study is that not all measures of biochemistry were assessed on the same day as the MR scan, hereby allowing natural day-to-day biological variations to affect the results. Additionally, most of the MR scans were performed after the start of intervention, which may have caused an underestimation of the ectopic fat reducing effect of treatment.

Furthermore, a large part of the children and adolescents assessed by MRS twice were excluded due to the 60 days limit criterion, why the presented data might be subject to a selection bias; e.g. that the study participants might have been more compliant to the treatment protocol than the excluded children and adolescents. Unfortunately, such exclusion is difficult to avoid in a study based on data from clinical practice, and the proposed time limit is important in order to justify concomitant changes within the data. Since the two groups were comparable in body composition both before and after treatment, we considered this selection bias acceptable.

Other limitations include that the sample size and the small changes in LFC may cause associations in regression analyses to be missed, and that the analyses of associations were made without adjusting for multiple testing, which increases the chance of type I errors. Furthermore, pubertal developmental stage was only assessed at baseline and not at follow-up.

## Conclusions

Reductions in magnetic resonance spectroscopy measured liver and muscle fat are attainable in multidisciplinary childhood obesity treatment, independent of the magnitude of weight loss and with concomitant improvements in lipid and glucose metabolism.

## Additional file

Additional file 1:
**STROBE statement for observational studies.** (DOC 85 kb)

## Abbreviations

BMI: body mass index
CI: confidence interval
HbA1c: glycosylated hemoglobin
HDL: high density lipoprotein
IMCL: intramyocellular lipids
MFC: muscle fat content
MR: magnetic resonance
MRI: magnetic resonance imaging
MRS: magnetic resonance spectroscopy
SAT: subcutaneous adipose tissue
SDS: standard deviation score
VAT: visceral adipose tissue
